# Elevated Atmospheric CO_2_ Modifies Symbiont-Associated Dispersal Behavior Under a Standardized Assay in an Aphid Clone

**DOI:** 10.3390/biology15191748

**Published:** 2026-10-02

**Authors:** Edward Mondor, Tyler Follman, Kerry M. Oliver

**Affiliations:** 1 Department of Biology, Georgia Southern University, Statesboro, GA 30458, USA; 2 Center for Forensic Sciences, Georgia Southern University, Statesboro, GA 30458, USA; 3 Department of Entomology, University of Georgia, Athens, GA 30602, USA

**Keywords:** *Acyrthosiphon pisum*, alarm pheromone, alarm signal, bottom-up effect, CO_2_, (*E*)-beta-farnesene, *Hamiltonella defensa*, top-down effect, tri-trophic interaction

## Abstract

Rising atmospheric CO_2_ levels may alter behavioral dynamics that mediate species interactions, a largely overlooked dimension of ecosystem functioning. Aphids offer a tractable model for examining these modifications, as their primary protection from natural enemies relies on the alarm pheromone (E)-β-farnesene (Eβf). Facultative aphid endosymbionts like Candidatus *Hamiltonella defensa* complicate this dynamic by modifying parasitoid resistance and associated behaviors, yet their interaction with elevated CO_2_ remains largely unexplored. Using a single clone of the pea aphid *Acyrthosiphon pisum* (Harris), we investigated how CO_2_ levels and *H. defensa* D3 status, independently and interactively, altered *Vicia faba* L. plant size, aphid fecundity, and aphid dispersal responses to Eβf. Here we show that dry shoot weights of bean plants grown under elevated CO_2_ increased, compared to those grown under ambient conditions. Irrespective of CO_2_ level, however, aphids harboring *H. defensa* produced significantly more offspring, and dispersed at significantly higher rates in response to Eβf than symbiont-free individuals. Under the standardized Eβf assay, the difference in dispersal between *H. defensa*-harboring aphids and those that were symbiont-free was large under ambient CO_2_ (59% vs. 9%) and smaller under elevated CO_2_ (23% vs. 12%). Thus, elevated CO_2_ reduced symbiont-associated differences in dispersal under standard assay conditions in this aphid clone with implications for tri-trophic interactions, biological control efficacy, and symbiont prevalence under future atmospheric conditions.

## Simple Summary

Elevated atmospheric carbon dioxide (CO_2_) modified symbiont-associated dispersal in aphids. While symbiont-carrying pea aphids produced almost twice as many offspring under all atmospheric conditions, under a standardized alarm pheromone (Eβf) assay, the difference in dispersal between symbiont-bearing and symbiont-free aphids was large under ambient CO_2_ and smaller under elevated CO_2_. Ultimately, rising emissions might change insect behaviors, potentially altering food webs and influencing biological control outcomes.

## 1. Introduction

Beyond its well-documented role in driving global temperatures, rising carbon dioxide (CO_2_) levels alter a wide range of biotic interactions [1,2]. From a pre-industrial level of approximately 280 ppm, atmospheric CO_2_ concentrations could reach as high as 1135 ppm by 2100 [3]. In terrestrial systems, elevated CO_2_ can alter plant resource dynamics, driving bottom-up effects on herbivore performance that vary by feeding guild [4,5,6,7,8,9,10]; for a comprehensive review on bottom-up effects, see Han et al. [11]. In legumes, which generally respond to atmospheric changes differently compared to other C3 crops due to symbiotic nitrogen fixation, elevated CO_2_ frequently stimulates photosynthesis, enhances aboveground biomass, and optimizes water-use efficiency, providing herbivores with a seemingly robust, healthy host [12,13]. These physiological changes, however, can alter the emission, diffusion, and reception of volatile organic compounds, including the emission and reception of alarm pheromones used by insects to communicate and evade natural enemies [14,15].

Aphids are a model system for studying chemical communication, which plays a central role in their interactions with predators and parasitoids [16,17]. The alarm pheromone (E)-β-farnesene (Eβf) is secreted through specialized abdominal structures (cornicles), signaling the presence of natural enemies to nearby conspecifics [18,19,20]. Aphids respond to Eβf in multiple ways, including immediate behaviors such as kicking, walking, or dropping from the host plant, and transgenerational responses such as increasing the proportion of winged offspring and/or choosing safer feeding positions [21,22,23]. Dropping incurs the greatest cost to aphid reproduction and survival [24,25] and depends on factors such as predation pressure, host plant characteristics, and the features of the environment into which the aphid would drop [26]. These dynamics are further complicated in the pea aphid, *Acyrthosiphon pisum* (Harris, 1776) (Hemiptera: Aphididae), which exists as a complex of host-specialized biotypes [27,28]. In this species complex, genetic variation among clones generates substantial diversity in Eβf responses both within and among populations [29,30].

Heritable variation in aphid-associated microbiota further shapes the diversity of alarm responses. The pea aphid harbors seven common facultative endosymbionts that are present in most sampled populations [31]. These symbionts mediate a diverse array of ecological interactions [32], including defense against thermal stress [33,34], parasitoids [35,36,37], and fungal and viral pathogens [38,39,40]. Unlike parasitoid resistance, direct symbiont-mediated protection against predators is not known to occur, as an effective deterrent would require the aphid to be rendered unpalatable or toxic prior to consumption [41]; however, certain symbionts can indirectly reduce predator fitness by impairing predator survival and development [42,43].

Symbionts may nonetheless modify behavioral responses to alarm pheromones. While pea aphids harboring *Serratia symbiotica* Sabri et al. 2011 (Enterobacterales: *Yersiniaceae*), Candidatus *Regiella insecticola* Moran et al. 2005 (Enterobacterales: *Yersiniaceae*) or *Rickettsia* da Rocha-Lima 1916 (Rickettsiales: *Rickettsiaceae*) were no more or less likely to drop from their host plants [44], those with Candidatus *Hamiltonella defensa* Moran et al. 2005 (Enterobacterales: *Yersiniaceae*) were less likely to display evasive behaviors in the presence of predators [45,46] (but see [30]). Furthermore, *H. defensa*-infected individuals exhibited increased reproduction following alarm pheromone exposure compared to symbiont-free aphids [47]. This suggests that rather than fleeing, *H. defensa*-harboring aphids may employ an alternative strategy of remaining on the host plant and increasing reproductive output. This behavior aligns with both the parasitoid resistance this symbiont confers [48] and the reduced fitness cost of staying put when natural enemies are detected [26].

In addition to biotic factors, abiotic conditions can also modify aphid life-history traits and alarm responses. While much has been learned about thermal stress on these interactions [49,50,51,52], rising atmospheric CO_2_ introduces a distinct set of physiological challenges [53,54,55]. Elevated CO_2_ can modify insect behavior directly, with previous studies showing that aphids under enriched atmospheres produce less Eβf and/or exhibit reduced dispersal responses to alarm pheromone [56,57,58]. Concurrently, elevated CO_2_ often alters host plant physiology, shifting biomass allocation between above- and below-ground tissues and modifying plant nutrient profiles [59,60]. These bottom-up effects could fundamentally alter the fitness of the herbivore, raising the critical question of how atmospheric conditions and microbial symbiosis interact to shape aphid reproduction and alarm responses in the context of ongoing climate change.

Building on these dynamics, we first quantified the effects of elevated CO_2_ on *Vicia faba* L. (Fabales: Fabaceae) shoot and root biomass. To test herbivore-level responses, we evaluated the performance and behavior of a single pea aphid clone harboring *H. defensa* strain D3 to address four key hypotheses: (1) pea aphids harboring the symbiont would produce fewer offspring than symbiont-free aphids under ambient conditions due to metabolic costs [61,62]; (2) symbiont-harboring aphids would disperse less frequently in response to alarm pheromone under ambient conditions; (3) both symbiont and non-symbiont-harboring aphids reared under elevated CO_2_ would exhibit reduced dispersal due to increased feeding resources; and (4) treatment effects would interact, such that aphids harboring *H. defensa* and reared under elevated CO_2_ would show the greatest reduction in alarm pheromone-induced dispersal.

Therefore, the aim of this study was to evaluate how a medium-high, end-of-century CO_2_ regime (842 ppm, consistent with SSP3-7.0) [3] interacts with the facultative symbiont *H. defensa* to alter aphid dynamics. Using a fully crossed experimental design, we quantified the combined effects of atmospheric enrichment and symbiont presence on plant growth and aphid ecology.

## 2. Materials and Methods

### 2.1. Insects

Clonal pea aphid lines were established using clone AS3, originally collected from *Medicago sativa* L. (Fabales: Fabaceae) in Logan County, Utah, USA, in 2007. This clone naturally harbors *H. defensa* strain D3 [63], which confers high levels of resistance to the most common parasitoid of pea aphids, *Aphidius ervi* Haliday, 1834 (Hymenoptera: Braconidae) [64,65]. A paired line carrying the same AS3 genotype, but lacking *H. defensa*, was generated by treating AS3 aphids with an antibiotic cocktail [66], providing an isogenic symbiont-free control.

Stock colonies of both lines, infected and uninfected, were maintained on broad bean (*V. faba* “Broad Windsor”) inside separate growth chambers at 24 °C under a 16:8 (L:D) photoperiod. Under these conditions, pea aphids reproduce via parthenogenesis, allowing genetically identical lines to be maintained indefinitely [67]. Colonies were maintained on staggered schedules and kept under strict environmental separation to prevent cross-contamination. Colonies were routinely genotyped to verify infection status.

### 2.2. Controlled Atmospheres

Atmospheric treatments were conducted inside a climate change system. The system comprised eight independent 29-gallon (76 cm long × 30 cm wide × 45 cm high) glass aquaria (Aqueon Inc., Franklin, WI, USA), covered by a clear Plexiglas sheet, and supplied by a centralized CO_2_ tank. Atmospheric regulation was maintained by two Sentinel Analyzers, model CHHC-4 (Global Product Solutions, Santa Rosa, CA, USA), running in “fuzzy-logic active” mode. One analyzer maintained four aquaria at an ambient baseline of 412 ± 12 ppm (*n* = 27), and one analyzer maintained four at an elevated CO_2_ level of 842 ± 20 ppm (*n* = 27) to simulate a medium-high end-of-century emissions scenario [3,68]. Internal gas levels were validated, and humidity levels controlled, using an electronically shielded Sentinel mobile sensor (Global Product Solutions, Santa Rosa, CA, USA), rotated randomly among the tanks. The same eight aquaria were thoroughly cleaned and re-used across trials. Illumination was provided by four ViparSpectra LED lights, model TC450 (ViparSpectra, Richmond, CA, USA). Each light measured 50 cm long × 21 cm wide × 7 cm high and supplied light to two aquaria. The lights were scheduled to operate on a 16:8 (L:D) photoperiod.

### 2.3. Plant Cultivation

All experimental plants were grown from seed within their designated ambient or elevated CO_2_ aquaria, with four to eight pots (2 seeds per pot) placed in each aquarium. Plants were grown in Miracle-Gro Moisture Control potting mix (The Scotts Company, Marysville, OH, USA), top-dressed with Osmocote 14-14-14 fertilizer (The Scotts Company, Marysville, OH, USA), and watered when the soil was dry, though all atmospheric treatments were watered simultaneously. Following emergence, seedlings were thinned to one per pot to reduce competition between plants. Experiments began approximately 7–9 days after planting, when all plants had at least one fully expanded leaf pair (not including the cotyledons).

### 2.4. Experiments

#### 2.4.1. Experiment 1: Plant Growth

**Figure 1:**
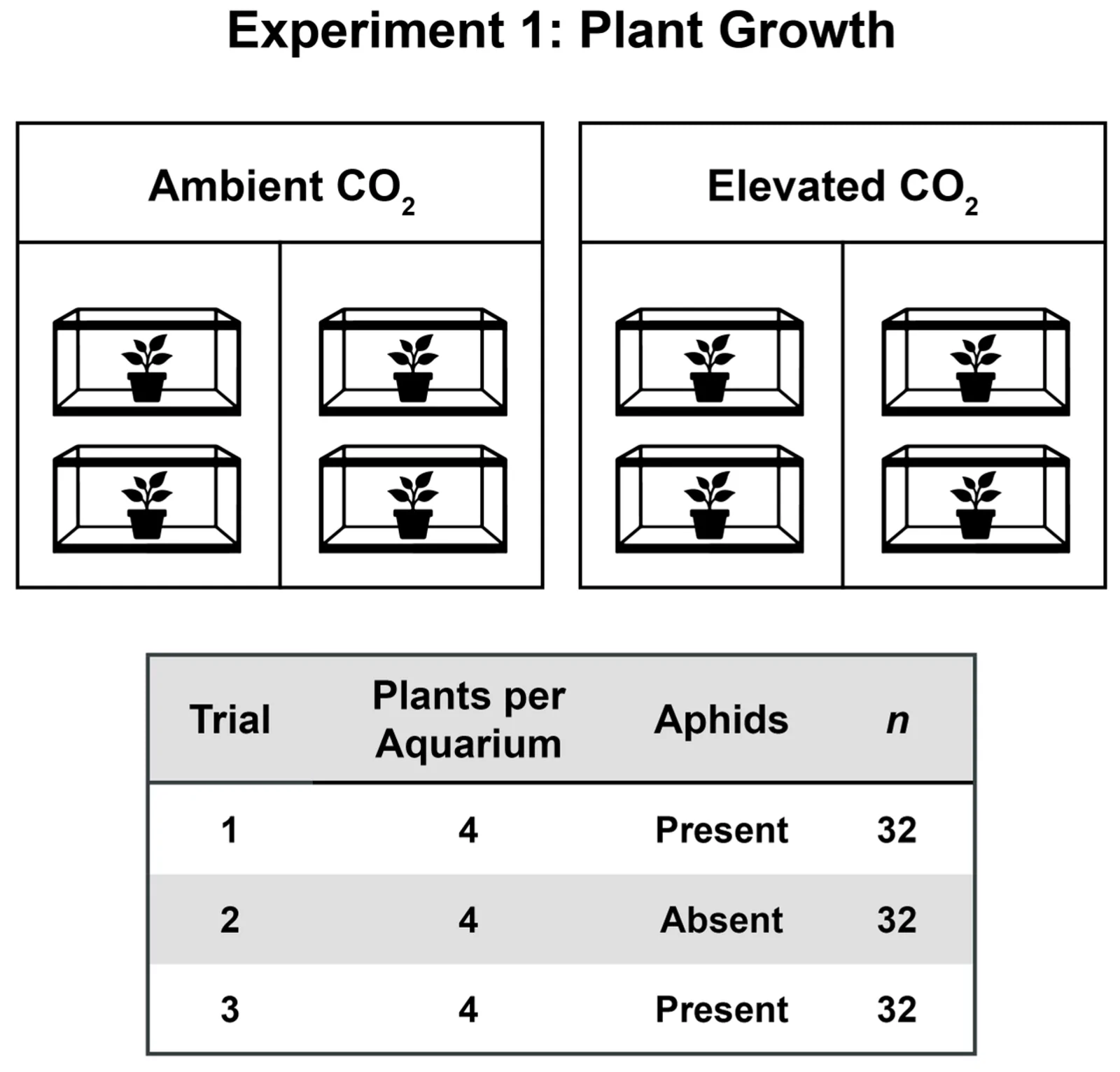
Experimental design and trial parameters for Experiment 1: Plant Growth. Schematic illustrates treatment chambers under ambient and elevated CO_2_ conditions. The table outlines trial specifications, including plant density, aphid presence or absence, and sample sizes.

To isolate the direct effects of elevated CO_2_ and aphid feeding on plant growth, mature, ~1-month-old *V. faba* plants from one aphid-free cohort (*n* = 32) and two aphid-infested cohorts (*n* = 32 each) were destructively harvested to quantify biomass allocation patterns (Figure 1). Above-ground vegetation (stems and leaves) was cut at the root-shoot transition zone at the soil level. Roots were gently washed in tap water to completely remove the potting soil. Tissue samples were placed in separate brown paper bags and placed in a drying oven at 40 °C for a minimum of 7 days. Samples were weighed on an analytical balance immediately upon removal from the dryer to determine shoot and root dry mass.

A small sub-sample (*n* = 3 ambient, 3 elevated) of these dried plant samples was subsequently homogenized and submitted to the University of Georgia Soil, Plant, and Water Laboratory for elemental macronutrient analysis.

#### 2.4.2. Experiment 2: Aphid Fecundity

To evaluate the effects of CO_2_ and symbiont status on aphid reproduction, plants were infested by placing four second-instar aphids from either the *H. defensa*-harboring or the symbiont-free stock colonies onto each plant using a fine paintbrush. Second-instar aphids were selected to maximize atmospheric exposure across telescoping generations while minimizing handling mortality. A mesh bag was placed around each pot, and secured with a rubber band, to restrict insect migration while allowing unrestricted gas exchange.

**Figure 2:**
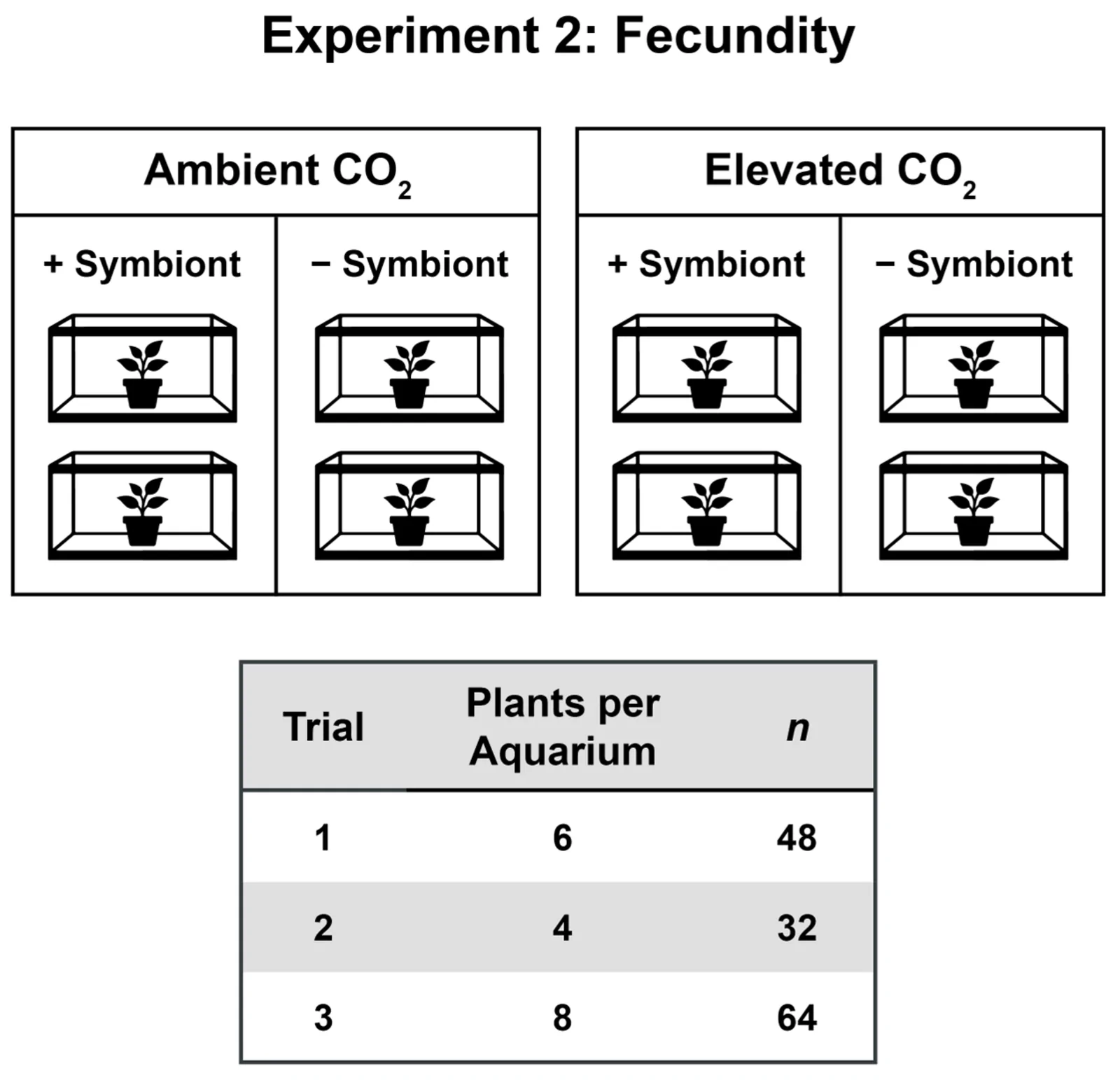
Experimental design and trial parameters for Experiment 2: Aphid Fecundity. Schematic depicts the 2 × 2 factorial setup combining CO_2_ level (ambient vs. elevated) and symbiont presence (+Symbiont vs. −Symbiont). The table summarizes trial specifications, including variations in plant density per aquarium and corresponding sample sizes.

For each of three trials (*n* = 48, 32, 64), aphids without symbionts were randomly assigned to two of the four ambient CO_2_ aquaria and two of the four elevated CO_2_ aquaria. Aphids with symbionts were assigned to the other two aquaria for each atmospheric treatment. This produced a fully crossed 2 × 2 factorial design (Figure 2). After allowing the aphids to feed and develop for one week, the number of adult aphids and the number of offspring were both recorded. Some aphids had failed to reach adulthood, so adult mortality was incorporated into this calculation to determine the average number of offspring per adult on each plant.

#### 2.4.3. Experiment 3: Aphid Behavior

To determine whether atmospheric treatments altered alarm responses, a randomized subset of the plants and aphid colonies from the fecundity experiment was used for the behavioral assays. After aphid counts were recorded in Experiment 2, adults were removed, and offspring were allowed to mature for an additional 3–4 days to reach the fourth instar. Escape responses were evaluated using synthetic alarm pheromone, Eβf (Penta Manufacturing, Livingston, NJ, USA). The treatment solution was prepared by diluting 0.6 µL of concentrated Eβf in 10 mL of hexane, yielding a concentration of approximately 50 ng/µL [21]; pure hexane served as the control. This specific Eβf concentration allows for direct comparison with previous studies [18,21], while maintaining biological relevance. As a single pea aphid attacked by a predator releases a mean of 16 ng, but a maximum of 49 ng, of Eβf [69], our applied dose is representative of levels given off by one to three attacked aphids.

**Figure 3:**
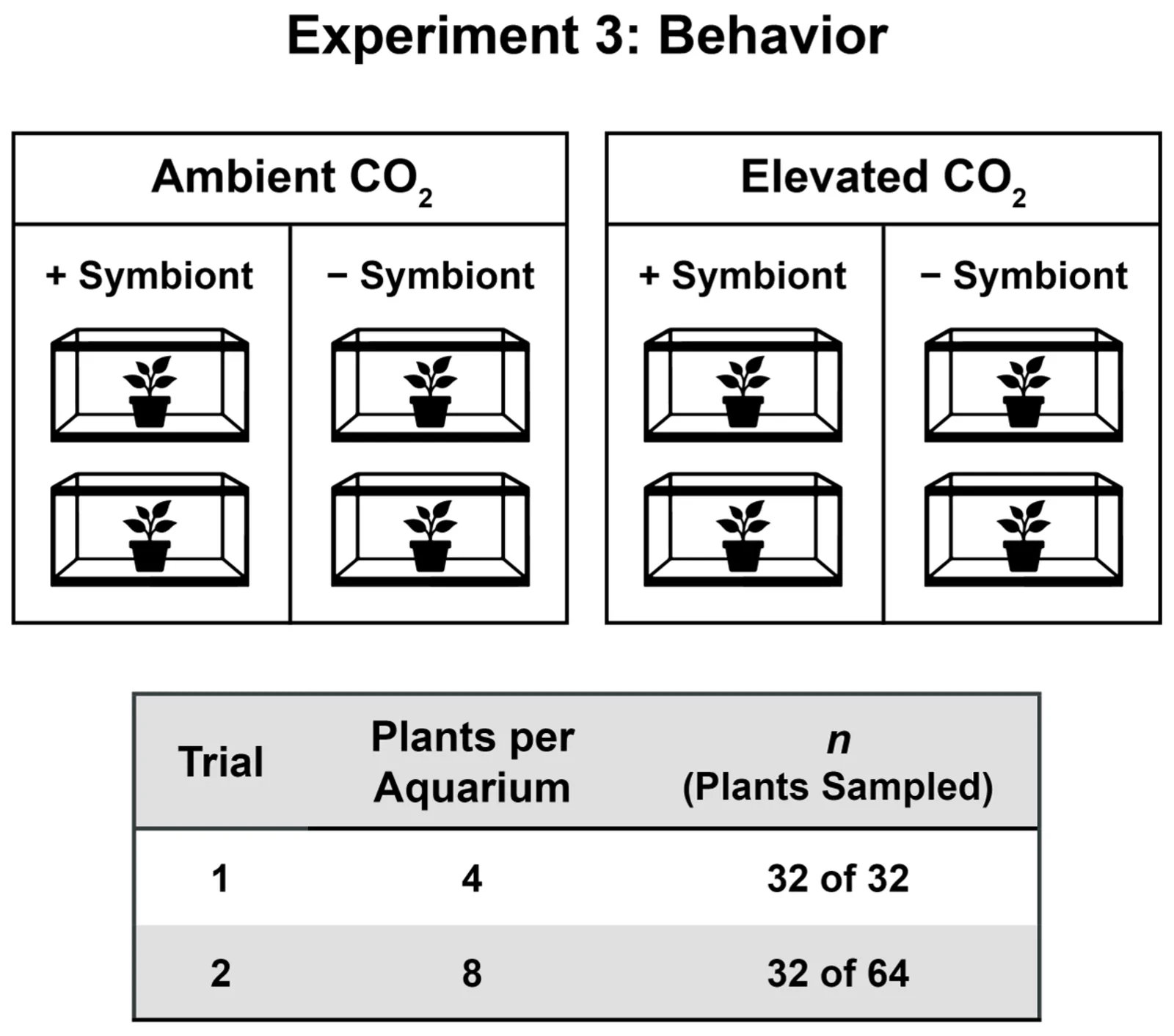
Experimental design and trial parameters for Experiment 3: Aphid Behavior. Schematic depicts the 2 × 2 factorial setup combining CO_2_ level (ambient vs. elevated) and symbiont presence (+Symbiont vs. −Symbiont). The table summarizes trial specifications, including variations in plant density per aquarium and the number of plants chosen for the experiments. Only one aphid cluster was examined per plant. Eight plants (six in Trial 1, two in Trial 2) were excluded because the aphids failed to form a distinct cluster.

From each of the four ambient and four elevated CO_2_ aquaria, two plants were randomly chosen for the Eβf treatment and two for the control. These plants were individually removed from the aquaria and immediately tested under standard room lighting and temperature conditions. (Figure 3). Only one cluster per plant was tested. Once a focal aphid cluster was identified on a plant, the number of aphids was counted. A 1 µL droplet of the designated solution was applied to a small piece of filter paper, which was held approximately 1 cm away from the cluster for 30 s using forceps. The number of aphids that dispersed out of the total cluster over those 30 s was recorded. Both behavioral trials (*n* = 32, 32) were video recorded to allow post hoc verification of dispersal counts. In trials 1 and 2, six and two plants, respectively, were excluded prior to analysis because aphids failed to form distinct clusters. Aphids had no prior exposure to Eβf or natural enemies prior to testing. Behavioral scoring was conducted unblinded.

### 2.5. Statistical Analyses

Statistical analyses were performed using JMP Student Edition 18.2.1 (JMP Statistical Discovery, Cary, NC, USA). Prior to testing, Shapiro–Wilk tests were conducted, and residual plots were checked for random scatter to confirm the underlying assumptions of normality and homoscedasticity. If necessary, data were transformed prior to parametric analyses (see details of the individual tests). All analyses were conducted using linear mixed models (LMMs) fitted via Restricted Maximum Likelihood (REML). To identify the aquarium as the true unit of replication (*n* = 4 per CO_2_ treatment level) and account for temporal variation across blocks, each aquarium was assigned a unique identity incorporating the trial number (e.g., Trial 1—Ambient 1, Trial 2—Ambient 1) and included as a random effect, thereby nesting plant subsamples within specific tanks and trials. The only exception was the plant growth analyses: because aphid infestation covaried with trial, trial was included as an additional random effect. Denominator degrees of freedom for all *F*-tests were calculated using the Kenward-Roger adjustment method in JMP, resulting in fractional degrees of freedom. When data transformation was required, statistical tests were performed on the transformed data, whereas figures display back-transformed values. For clarity, proportional data are presented as percentages in both the text and figures.

#### 2.5.1. Experiment 1: Plant Growth

In the LMM, fixed effects included CO_2_ level (ambient vs. elevated), aphid infestation (absent vs. present), and their interaction. Aquarium identity and trial (nested within aphid infestation status) were both entered as random effects. The dependent variables, analyzed separately, were shoot dry weight (g), root dry weight (g), and shoot/root ratio.

#### 2.5.2. Experiment 2: Aphid Fecundity

In the LMM, main effects in the model were CO_2_ (ambient vs. elevated), symbiont (absent vs. present), and the CO_2_ × symbiont interaction. Aquarium identity was entered as a random effect. The dependent variable, number of offspring per adult, was transformed using natural log (ln) to fit the assumption of normality, prior to analysis.

#### 2.5.3. Experiment 3: Aphid Behavior

In the LMM, main effects included CO_2_ level (ambient vs. elevated), symbiont status (absent vs. present), pheromone treatment (hexane control vs. Eβf), and all two- and three-way interactions. Aquarium identity was entered as a random effect. The dependent variable, the proportion of aphids per cluster that dispersed, was arcsine square-root transformed prior to analysis to satisfy normality assumptions. Due to the extremely low responses to the hexane control, LSMeans contrasts for only the Eβf treatment were conducted post hoc.

## 3. Results

### 3.1. Experiment 1: Plant Growth

There were differences in vegetative investment strategies under altered atmospheric environments. Above-ground shoot mass was approximately 18% greater under enhanced CO_2_ (mean ± 1 se; ambient = 1.17 ± 0.13 g, elevated = 1.37 ± 0.13 g) (F_1,7.1_ = 5.72, *p* = 0.048). Above-ground biomass was not significantly different if it was aphid-infested (mean ± 1 se; absent = 1.34 ± 0.19 g, present = 1.20 ± 0.14 g) (F_1,1.0_ = 0.39, *p* = 0.64). There was no significant first-order interaction (F_1,85_ = 0.28, *p* = 0.60). Neither the random effects of aquarium (variance ratio = 0.036, Wald *p* = 0.61) nor trial (variance ratio = 0.29, Wald *p* = 0.52) were significant.

Below-ground root mass was not significantly influenced by CO_2_ level (mean ± 1 se; ambient = 1.20 ± 0.18 g, elevated = 1.28 ± 0.18 g) (F_1,7.0_ = 0.73, *p* = 0.42) or aphid-infestation (mean ± 1 se; absent = 1.42 ± 0.28 g, present = 1.06 ± 0.20 g) (F_1,1.0_ = 1.08, *p* = 0.49). There was no significant first-order interaction (F_1,85.0_ = 0.035, *p* = 0.85). The random effects of aquarium (variance ratio = 0.042, Wald *p* = 0.57) and trial (variance ratio = 0.53, Wald *p* = 0.50) were also not significant.

The proportional allocation between shoots and roots did not shift significantly based on CO_2_ (mean ± 1 se; ambient = 1.06 ± 0.34, elevated = 1.23 ± 0.34) (F_1,7.4_ = 2.97, *p* = 0.13). Shoot/root ratios were not significantly smaller if plants were aphid-infested (mean ± 1 se; absent = 1.01 ± 0.55, present = 1.27 ± 0.39) (F_1,1.0_ = 0.14, *p* = 0.77). There was no significant first-order interaction (F_1,85.0_ = 0.77, *p* = 0.38). The random effects of aquarium (variance ratio = 0.011, Wald *p* = 0.85) and trial (variance ratio = 1.58, Wald *p* = 0.49) were not significant.

While sample sizes were insufficient to conduct statistical analyses on leaf tissue chemistry, foliar concentrations of the key macronutrients nitrogen, phosphorus, potassium, and sulfur appeared to remain stable, though this cannot be verified without additional testing. This suggests that the host plants may have been similar under atmospheric enrichment. The structural secondary macronutrients calcium and magnesium exhibited an upward trend (Table 1).

### 3.2. Experiment 2: Aphid Fecundity

**Figure 4:**
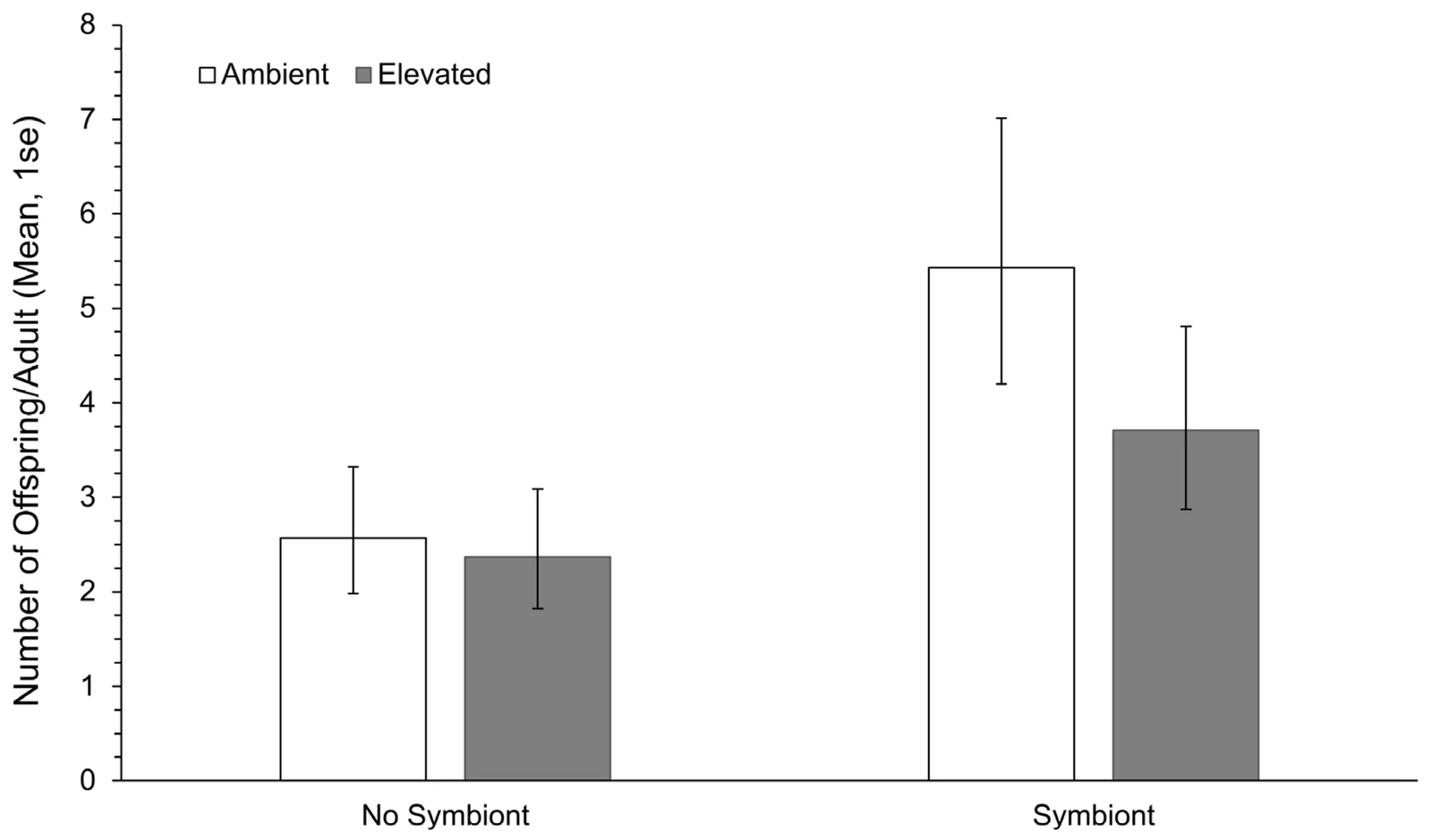
Mean number of offspring born per adult pea aphid over 1 week, under ambient and elevated CO_2_ with and without *Hamiltonella defensa* symbionts. Values are back-transformed LSMeans, 1 standard error (*n* = 4 aquaria per CO_2_ level, representing *n* = 2 aquaria per CO_2_ × symbiont treatment combination per trial) analyzed using a linear mixed model (LMM) with aquarium identity as a random effect. Offspring production was significantly affected by symbiont presence (F_1,20.9_ = 5.33, *p* = 0.031), but not CO_2_ (F_1,20.9_ = 0.79, *p* = 0.39). There was no CO_2_ × symbiont interaction (F_1,20.9_ = 0.33, *p* = 0.57).

The CO_2_ levels did not significantly affect reproductive output, as adult aphids produced a similar number of offspring under both ambient and elevated atmospheric conditions (mean, 1 se; ambient = 3.73 (3.11–4.48), elevated = 2.97 (2.46–3.57)) (F_1,20.9_ = 0.79, *p* = 0.39). In contrast, symbionts largely affected aphid fitness, as those individuals harboring *H. defensa* produced almost twice as many offspring as those without the symbiont (mean, 1 se; no symbiont = 2.47 (2.05–2.97), symbiont = 4.49 (3.74–5.39)) (F_1,20.9_ = 5.33, *p* = 0.031). No significant first-order interaction was observed between CO_2_ level and symbiont presence, indicating that the reproductive advantage conferred by *H. defensa* remained consistent across both present-day and future atmospheric conditions (F_1,20.9_ = 0.33, *p* = 0.57) (Figure 4). The random effect of aquarium was significant (variance ratio = 0.64, Wald *p* = 0.016).

### 3.3. Experiment 3: Aphid Behavior

**Figure 5:**
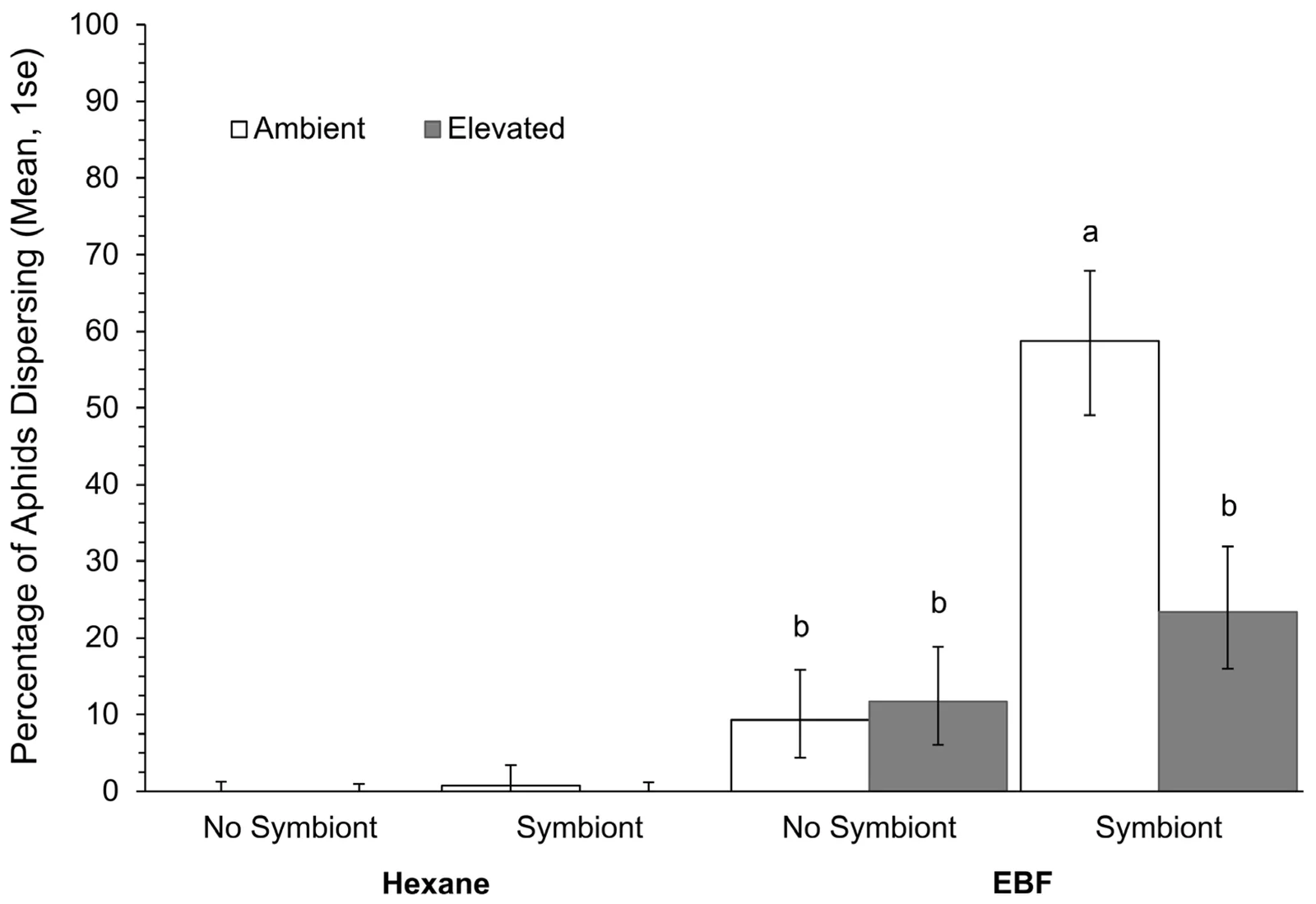
Percentage of pea aphids dispersing in response to hexane and alarm pheromone when harboring and not harboring the symbiont *Hamiltonella defensa* in ambient vs. elevated CO_2_ atmospheres. Values are back transformed LSMeans, 1 standard error (*n* = 4 aquaria per CO_2_ level, representing *n* = 2 aquaria per CO_2_ × symbiont treatment combination per trial) analyzed using a linear mixed model (LMM) with aquarium identity as a random effect. There was no significant CO_2_ × symbiont × pheromone treatment interaction (F_1,38.1_ = 1.24, *p* = 0.27), however, letters over the bars for the Eβf treatment indicate significant differences using LSMeans contrasts (bars with different letters are significantly different; *p* < 0.05).

The main effect of CO_2_ did not significantly affect dispersal rates (mean, 1 se; ambient = 9.76 (6.99–13.1)%, elevated = 4.45 (2.64–6.82)%) (F_1,10.9_ = 2.32, *p* = 0.16). In contrast, symbiont status had a significant effect on behavior; aphids harboring *H. defensa* dispersed at higher rates than symbiont-free aphids (mean, 1 se; no symbiont = 2.67 (1.32–4.54)%, symbiont = 12.79 (9.78–16.27)%) (F_1,10.9_ = 8.48, *p* = 0.014). Confirming the effectiveness of our pheromone bioassay, aphids dispersed when exposed to Eβf, whereas almost no response was observed to the hexane control (mean, 1 se; hexane = 0.04 (0.00–0.54)%, Eβf = 23.75 (19.74–28.11)%) (F_1,38.1_ = 44.10, *p* < 0.0001). The only significant first-order interaction was between symbiont presence and pheromone treatment (F_1,38.1_ = 4.66, *p* = 0.037). There were no significant first-order interactions between CO_2_ and symbiont presence (F_1,10.9_ = 3.09, *p* = 0.11), nor CO_2_ and pheromone treatment (F_1,38.1_ = 0.64, *p* = 0.43). The third-order interaction between CO_2_, symbiont presence, and pheromone treatment was not statistically significant (F_1,38.1_ = 1.24, *p* = 0.27). Given the low baseline response to the hexane control, LSMeans contrasts were evaluated within the Eβf treatment. Under ambient CO_2_, symbiont status strongly influenced dispersal following Eβf exposure. Only 9.27% of symbiont-free aphids dispersed, compared to 58.73% for *H. defensa*-harboring individuals (F_1,33.95_ = 16.99, *p* = 0.0002). This behavior was drastically reduced under elevated CO_2_, however, for those harboring the symbiont (symbiont-free: 11.67%; *H. defensa*: 23.43%) (F_1,33.87_ = 1.31, *p* = 0.26) (Figure 5). The random effect of aquarium was not significant (variance ratio = −0.034, Wald *p* = 0.78).

## 4. Discussion

Understanding how elevated CO_2_ shapes insect chemical communication is increasingly important as atmospheric greenhouse gases continue to rise and their ecological consequences compound [14,15]. Symbiotic associations are thought to be among the interactions most vulnerable to climate change, yet symbionts may also facilitate rapid host adaptation to novel environments [52,70,71,72,73,74]. Aphids and their facultative endosymbionts represent a particularly informative system for examining how these forces interact, as aphid responses to natural enemies depend largely on the production and reception of the alarm pheromone Eβf, a system sensitive to elevated CO_2_ [56,58]. Facultative symbionts, such as *H. defensa*, can modify parasitoid resistance, alarm pheromone production, and related behaviors [46,47,48,75]. We tested whether *H. defensa* status and CO_2_ level, independently and in combination, altered host plant growth, as well as pea aphid fecundity and dispersal responses to Eβf in a standardized assay. Our results revealed that elevated CO_2_ increased plant size, while *H. defensa* infection significantly increased aphid fecundity and dispersal. Elevated CO_2_, however, reduced the symbiont-associated dispersal observed under ambient conditions.

A single clone of pea aphid harboring *H. defensa* strain D3 exhibited a large reproductive advantage, producing almost twice as many offspring as those that were symbiont-free, across both atmospheric treatments. This finding directly refutes our hypothesis that the metabolic costs of maintaining the symbiont would reduce host fecundity. While facultative symbionts are generally associated with fitness trade-offs in the absence of natural enemies [76], certain protective strains have been shown to provide physiological or nutritional compensations that mitigate these costs depending on the host clonal background [62,77]. In our system, the substantial increase in fecundity suggests that *H. defensa* strain D3 may actively manipulate host reproductive physiology or improve nutrient assimilation on *V. faba*, at least in this one clone. Crucially, this reproductive advantage remains intact under future atmospheric conditions.

Contrary to our second hypothesis, aphids harboring *H. defensa* dispersed at significantly higher rates than symbiont-free aphids; a result that contrasts with prior reports of reduced alarm pheromone production and evasive behavior in *H. defensa*-harboring aphids [45,46,75,78]. One potential explanation involves the pheromone dose used in our bioassay. The concentration used here was approximately one to three times the amount produced by a single pea aphid [69], which may have been perceived as multiple individuals being attacked simultaneously [79,80]. If *H. defensa*-harboring aphids employ a threshold-dependent response strategy, as has previously been shown in wing-induction responses [81], they may choose to remain on the plant when Eβf concentrations are low, relying on the highly effective immune protection the symbiont provides against single parasitoids, but disperse rapidly when concentrations signal a broader or more severe attack from natural enemies that the symbiont cannot neutralize. Under this hypothesis, the elevated dispersal we observed may reflect behavioral responses near the high end of that sensitivity curve. Aphids may also respond differently to an actively searching predator or parasitoid as opposed to a solitary Eβf emission, as extreme alarm responses like dropping incur steep fitness costs [24,25]. Further experimentation is required, however, to determine if different Eβf concentrations produce different aphid responses, and whether live natural enemies generate different behavioral outcomes.

This interpretation is consistent with the enemy-specific nature of symbiont-mediated defense [46,82]: parasitoid resistance reduces the fitness cost of remaining in place under low-threat conditions, whereas threats that *H. defensa* cannot physically protect against should trigger stronger dispersal. Central to this hypothesis is whether *H. defensa* modifies an aphid’s neurosensory sensitivity to Eβf. Elevated CO_2_ has been shown to reduce alarm responses in the grain aphid *Sitobion avenae* (Fabricius, 1775) (Hemiptera: Aphididae) [83] and in an aphid feeding on aspen poplar, *Chaitophorus stevensis* Sanborn, 1904 (Hemiptera: Aphididae) [58], suggesting that reception may be sensitive to atmospheric shifts. Whether *H. defensa* similarly alters Eβf sensitivity, and how that threshold is further modified by elevated CO_2_, remains unresolved. Future work using live natural enemies across a range of densities and pheromone gradients would help disentangle production and reception effects.

A second possibility is that *H. defensa* effects on Eβf-mediated dispersal vary among microbial strains. Just as distinct strains differ considerably in the strength of protection conferred and the metabolic costs imposed on their hosts [48], they may also differ in their downstream influence on alarm pheromone sensitivity and/or behavioral thresholds. Symbiont strain-by-host genotype interactions could further complicate this picture, as clonal genetic variation in Eβf responses has been documented in the literature [29,30]. The present study used a single aphid clone paired with a single *H. defensa* strain, which precludes broad conclusions about the generality of these findings. Broader studies incorporating multiple host clones and symbiont strains, and those involving other insect species [84], would help identify strain-specific effects from those attributable to *H. defensa* infection more generally.

Our third hypothesis, that aphids reared under elevated CO_2_ would exhibit a significant reduction in dispersal, was not supported as a main effect. Although fewer aphids dispersed under atmospheric enrichment, the main effect was not statistically significant. This finding contrasts with prior studies that did not account for symbiont status [56,58]. As noted above, our bioassay used a standardized exogenous Eβf dose rather than relying on endogenous pheromone release from an aphid. Hence, if the primary effect of elevated CO_2_ on field aphids operates by suppressing Eβf emission rather than impairing antennal Eβf sensitivity, a fixed exogenous dose would produce similar dispersal responses across both atmospheric treatments. This distinction has practical implications for interpreting field responses to climate change, where both production and reception pathways operate simultaneously.

Prior studies frequently attribute altered herbivore behavior under elevated CO_2_ to bottom-up nutritional decline; however, our data suggest that foliar macronutrient levels (N, P, K, and S) did not differ widely between ambient and elevated treatments. With the significantly enhanced aboveground shoot biomass, our results suggest that the host plants may have remained healthy and nutritionally stable [85]. The slight directional drops in macronutrients may represent a slight growth dilution, where rapid carbon accumulation outpaces mineral uptake in thriving tissues, rather than a deficiency, per se [13,59,86]. This view, while speculative as it is based on an extremely low sample size, is supported by the concurrent increases in calcium and magnesium, structural minerals driven passively into the plant’s tissue by transpiration [87,88]. More experiments on plant nutrition, however, would be required to reach any firm conclusions.

Because the host plants were larger under elevated CO_2_, they may have provided a good quality resource to the aphids feeding on them. Dispersing from a host plant to escape danger is well known to be a risky and energetically costly response [25]. When occupying a potentially high-quality host, the evolutionary cost of abandoning the plant increases dramatically [24]. While our elemental analysis showed that the plants may have been similar, it did not directly evaluate phloem composition or plant defenses. Atmospheric enrichment, however, frequently causes a growth dilution effect that can suppress defensive secondary compounds [89]. If defensive metabolites are diluted under elevated CO_2_, the metabolic cost of ingestion and detoxification would drop. We hypothesize that for symbiont-harboring aphids this behavioral difference is fundamentally driven by host value; the combined reward of exploiting a vigorous, and potentially less defended, plant overrides the perceived threat of the alarm pheromone signal. Remaining on the plant is an opportunity to exploit a thriving environment. A comprehensive nutrient and phloem analysis would be required to test this hypothesis.

Our last hypothesis was partially supported. Because the three-way interaction (CO_2_ × symbiont × pheromone) was not statistically significant (*p* = 0.27), our data do not establish a treatment-by-pheromone interaction of a selective disruption of chemical alarm communication per se. Conditional contrasts, however, within the standardized Eβf assay demonstrated that the difference in dispersal between symbiont-bearing and symbiont-free aphids was large under ambient CO_2_ (59% vs. 9%), and not significantly different under elevated CO_2_ (23% vs. 12%). This behavioral convergence suggests that elevated CO_2_ reduces symbiont-associated differences in dispersal under standard assay conditions. Rather than reflecting a host-mediated asset trade-off, this behavioral convergence may be driven by direct physiological constraints on the symbiont. For example, atmospheric enrichment alters the pH of insect hemolymph and gas exchange dynamics, environments where *H. defensa* resides [74,90]. Such physiological stress could suppress symbiont titers within the host, effectively reducing symbiont-mediated dispersal toward the symbiont-free baseline. If elevated CO_2_ routinely reduces this behavioral responsiveness under future atmospheric conditions, *H. defensa*-harboring aphids may face heightened vulnerability to generalist predators, shifting the evolutionary benefit of maintaining these microbes in wild populations. Until *H. defensa* density or localization is quantified, that is, measuring symbiont titer using qPCR, their relative abundance under different CO_2_ conditions, and host insect physiological changes associated with symbiont maintenance, the mechanistic basis remains unknown. Whether comparable interactions occur with other aphid facultative symbionts also remains an open question.

Several additional directions would strengthen the interpretation of these multi-trophic findings. Future studies should assess whether specific instars modulate dispersal responses, as age-related variation in antipredator behavior is common in aphids [91]. More broadly, these results highlight the need to integrate microbial ecology into climate change models. Doing so is essential for predicting shifts in insect behavior and tri-trophic interactions, which ultimately may influence future predator-prey interactions and biological control outcomes.

## 5. Conclusions

Rising atmospheric CO_2_ levels have the potential to influence symbiont-associated behavioral dynamics in insect populations. While the facultative symbiont *H. defensa* confers a robust reproductive advantage to this pea aphid clone regardless of atmospheric conditions, under a standardized Eβf assay, the difference in dispersal between symbiont-bearing and symbiont-free aphids was large under ambient CO_2_ and smaller under elevated CO_2_. By dampening these symbiont-associated dispersal differences, enriched CO_2_ environments may alter ecological outcomes for symbiont-harboring individuals, leaving these populations increasingly vulnerable to generalist predators, shifting the evolutionary cost–benefit dynamics of maintaining these protective microbes.

## Figures and Tables

**Table 1:** Mean percentages (±1 se) of macronutrients in above-ground dried plant tissue grown under ambient (*n* = 3) and elevated (*n* = 3) atmospheric CO_2_. Macronutrients are ordered by primary components (N, P, K) followed by secondary structural elements (Ca, Mg, S).

Atmosphere	Nitrogen (N)	Phosphorus (P)	Potassium (K)	Calcium (Ca)	Magnesium (Mg)	Sulfur (S)
Ambient	5.36 (0.69)	0.55 (0.13)	4.15 (0.80)	0.70 (0.05)	0.82 (0.08)	0.40 (0.02)
Elevated	5.02 (0.44)	0.48 (0.16)	3.89 (0.86)	0.84 (0.21)	0.92 (0.20)	0.33 (0.03)

## Data Availability

The data presented in this study are available upon reasonable request from the corresponding author.
